# Intra‐individual variation of circulating tumour DNA in lung cancer patients

**DOI:** 10.1002/1878-0261.12546

**Published:** 2019-08-16

**Authors:** Johanne A. Hojbjerg, Anne T. Madsen, Hjordis H. Schmidt, Steffen F. Sorensen, Magnus Stougaard, Peter Meldgaard, Boe S. Sorensen

**Affiliations:** ^1^ Department of Clinical Biochemistry Aarhus University Hospital Denmark; ^2^ Department of Oncology Aarhus University Hospital Denmark; ^3^ Department of Pathology Aarhus University Hospital Denmark

**Keywords:** circulating tumour DNA, digital PCR, Intra‐individual variation, lung cancer, next‐generation sequencing

## Abstract

Circulating tumour DNA (ctDNA) has been increasingly incorporated into the treatment of cancer patients. ctDNA is generally accepted as a powerful diagnostic tool, whereas the utility of ctDNA to monitor disease activity needs to be fully validated. Central to this challenge is the question of whether changes in longitudinal ctDNA measurements reflect disease activity or merely biological variation. Thus, the aim of this study was to explore the intra‐individual biological variation of ctDNA in lung cancer patients. We identified tumour‐specific mutations using next‐generation sequencing. Day‐to‐day and hour‐to‐hour variations in plasma concentrations of the mutant allele and wild‐type cell‐free DNA (cfDNA) were determined using digital PCR. The levels of the mutant alleles varied by as much as 53% from day to day and 27% from hour to hour. cfDNA varied up to 19% from day to day and up to 56% from hour to hour, as determined using digital PCR. Variations were independent of the concentration. Both mutant allele concentrations and wild‐type cfDNA concentrations showed considerable intra‐individual variation in lung cancer patients with nonprogressive disease. This pronounced biological variation of the circulating DNA should be investigated further to determine whether ctDNA can be used for monitoring cancer activity.

**Abbreviations**
cfDNA: cell‐free DNA
CT: computed tomography
ctDNA: circulating tumour DNA
CV: coefficient of variation
ddPCR: droplet digital PCR
EGFR: epidermal growth factor receptor
LoD: limit of detection
NGS: next‐generation sequencing
SCLC: small cell lung cancer

## 1 Introduction

Tumour DNA genotyping is becoming the standard practice in the treatment of cancer (Hench, Hench and Tolnay, 2018). Genotyping is necessary to detect the genetic alterations responsive to the targeted treatments. For instance, mutations in the epidermal growth factor receptor (*EGFR*) predict the response to tyrosine kinase inhibitors in lung cancer (Mok *et al.*, 2009; Paez *et al.*, 2004) and the genotyping of the *EGFR* in plasma is validated and approved as a diagnostic implement in the clinical setting (Planchard *et al.*, 2018; U.S. Food and Drug Administration (2016).

By targeting specific gene alterations, cancer therapy is moving towards a more personalised approach (Oellerich *et al.*, 2017). Since it can be challenging in some cases to obtain the tissue suitable for genetic analysis (Fenizia *et al.*, 2015), circulating tumour DNA (ctDNA) has been studied intensively as a way to gain genetic information in a less invasive way (Oxnard *et al.*, 2014).

Circulating tumour DNA is currently implemented as a diagnostic tool, but ctDNA also holds tremendous potential as a biomarker for monitoring cancer evolution and disease activity (Murtaza *et al.*, 2015; Murtaza *et al.*, 2013; Oellerich *et al.*, 2017). The short half‐life of ctDNA (~ 2 h) (Lo *et al.*, 1999; Yu *et al.*, 2013) makes real‐time molecular monitoring of cancer possible. Several publications have shown that ctDNA dynamics reflect tumour activity (Diehl *et al.*, 2008; Sorensen *et al.*, 2014). Furthermore, if ctDNA is present after curative treatment, the risk of relapse is significantly increased (Garcia‐Murillas *et al.*, 2015). Likewise, an increase in the concentration of ctDNA during ongoing treatment precedes disease progression (Demuth *et al.*, 2018; Provencio *et al.*, 2017; Xiong *et al.*, 2017). These findings offer new hope for monitoring disease activity using ctDNA and emphasise how ctDNA may be a valuable tool in the treatment–decision pathway in the near future.

However, the application of ctDNA as a monitoring biomarker places further demands on the quality of the ctDNA analysis (Campos *et al.*, 2018). Monitoring requires the exact quantification of ctDNA in order to accurately reflect cancer activity. The percentage of ctDNA in proportion to wild‐type cell‐free DNA (cfDNA), termed the allele frequency, is often used to adjust for the total amount of DNA in the sample. Hence, the intra‐individual biological variation of ctDNA and wild‐type cfDNA in nonprogressive disease must be clarified before ctDNA dynamics should be used to determine disease activity. Knowledge of the underlying biological variance is essential to define a clinically significant change in ctDNA. Before making treatment decisions based on ctDNA dynamics, we have to ensure that the registered fluctuations are reflecting disease activity and not just natural biological variations. However, knowledge of ctDNA dynamics in cancer patients with nonprogressive disease is virtually absent.

Thus, the aim of the present study was to explore the intra‐individual biological variation of ctDNA concentrations in lung cancer patients with nonprogressive disease. We analysed the mutant allele concentrations from hour to hour and from day to day in 11 lung cancer patients with radiologically stable or responsive disease and undergoing no current treatment.

## 2 Materials and methods

### 2.1 Study design and patients

Patients with stage IV lung cancer from the Department of Oncology, Aarhus University Hospital, Denmark, were included in a clinical prospective observational study between May 2016 and June 2018. Included patients met the following criteria: (a) advanced lung cancer; (b) response or stable disease by the response evaluation criteria in solid tumours criteria (Eisenhauer *et al.*, 2009) on the latest computed tomography (CT) scan; (c) no current anticancer treatment; and (d) aged above 18. Patients provided written informed consent before inclusion.

The exclusion criteria were as follows: (a) active cancer other than lung cancer; and (b) infection.

The study was approved by the Committees on Health Research Ethics of the Region of Central Denmark (record number: 1‐10‐72‐55‐15) and the Danish Data Protection Agency (record number: 1‐16‐02‐231‐15). The study was conducted according to the Declaration of Helsinki principles.

### 2.2 Laboratory analyses

#### 2.2.1 Sampling

Blood samples were collected in EDTA tubes over three successive days at the same time each day. Two samples with a 1‐h interval were collected on the first and second day. Only one sample was taken on the third day amounting to a total of five blood samples for each patient. Ten milliliter of EDTA blood was collected at each time point resulting in a total amount of 50 mL EDTA blood from each patient. The patients were instructed to rest between sampling. The EDTA blood was centrifuged for 30 min after collection at 1850 ***g*** for 9 min and was subsequently frozen at −80 °C until DNA extraction.

#### 2.2.2 DNA extraction from plasma

The plasma was thawed on ice and centrifuged at 13 000 ***g*** for 10 min prior to DNA extraction (van Ginkel *et al.*, 2017). DNA was extracted from between 2.8 and 4 mL of EDTA plasma using the QIAamp® Circulating Nucleic Acid kit (Qiagen, Hilden, Germany) according to the manufacturer's protocol. Results were subsequently adjusted for the amount of plasma from which cfDNA was extracted. The coefficient of variation (CV) of the extraction was determined to 6–10% (data not shown). CfDNA was eluted in 100 μL of elution buffer. The DNA concentration was determined by Qubit® 2.0 (Invitrogen by Life Technologies Corporation, Eugene, OR, USA). The size of the extracted cfDNA was assessed using the High Sensitivity DNA kit on an Agilent 2100 Bioanalyzer system (Santa Clara, CA, USA) according to the protocol provided by the manufacturer.

#### 2.2.3 DNA extraction from the buffy coat

The buffy coat was examined to rule out a germline mutation when a mutation was discovered in plasma, and the matching tissue was unavailable. The DNA was purified from the 200 μL buffy coat using a QIAamp® DNA Blood Mini Kit (Qiagen) according to the manufacturer's protocol.

#### 2.2.4 Sequencing

Next‐generation sequencing (NGS) of plasma was used to identify the mutations suitable for repeated measurements by droplet digital PCR (ddPCR) in the patients without available tumour tissue.

Sequencing libraries were prepared from cfDNA (2.8–9.7 ng) using the Oncomine™ Solid Tumour DNA kit (OST; Thermo Fisher Scientific, Watham, MA, USA) as described previously (Winther‐Larsen *et al.*, 2017). In short, the Ion Chef™ Instrument (Thermo Fisher Scientific) was used for sample preparation, and the sequencing was conducted using the Ion Personal Genome Machine® (PGM™; Thermo Fisher Scientific, Watham, MA, USA) System. The Ion 316™ v2 BC chips were loaded with four samples. Variant calling was performed using the ion reporter Software (version 5.4; Thermo Fisher Scientific, Watham, MA, USA) and the AmpliSeq CHPv2 peripheral/CTC/CF DNA single sample workflow (Thermo Fisher Scientific). The samples were included whether the mean depth reached ≥ 2000. The variants were called if they were reported to COSMIC and the allele frequency ≥ 1%. The Integrative Genomics Viewer v.2.3.77 (Broad Institute, Cambridge, MA, USA) was used for the manual visualisation of variants (Robinson *et al.*, 2011).

#### 2.2.5 Droplet digital PCR

Droplet digital PCR was performed using the QX200™ AutoDG™ Droplet Digital™ PCR System (Bio‐Rad, Hercules, CA, USA). The reaction volume was 22 µL and consisted of 2× Supermix for probes (no UTP), 900 nm primers, 250 nm probes, and 9 µL of purified cfDNA. All samples were conducted in triplicate [with an intra‐run variability of 2.8–3.6% (data not shown)] as a minimum to avoid any variance caused by subsampling. All five samples from each patient were analysed in the same run to avoid run‐to‐run variability. All assays and reagents were purchased from Bio‐Rad. Wet‐lab validated assays were used when possible, and the remaining assays were designed by Bio‐Rad and validated in‐lab. The limit of detection (LoD) for each assay was determined using blood samples from anonymous donors collected from the blood bank at the Aarhus University Hospital as previously described (Milbury *et al.*, 2014). LoD and assay information can be found in Table S1.

Data were analysed using quantasoft v.1.7.4.0917 software (Bio‐Rad). Each run contained positive and negative controls. Gene Strands (Eurofins Genomics, Ebersberg, Germany) diluted in cfDNA from anonymous blood donors were used as mutation‐positive controls. Results were reported as copies per mL plasma and calculated by the following equation:

$$\text{Copies}/\mu \text{L}\left(\text{reaction}\right)\times 22\mu \text{L}/\text{cfDNA}\;\mu \text{L}\left(\text{input}\right)\times 100\mu \text{L}\left(\text{elution volume}\right)/\text{mL plasma input}.$$

#### 2.2.6 Tissue

The Oncomine™ Solid Tumour DNA kit (OST; Thermo Fisher Scientific) was used to determine the mutation status in the diagnostic biopsies by NGS.

### 2.3 Statistics

The CV% was chosen to measure the intra‐individual plasma DNA variation. Statistical analysis was performed using prism version 7.0b (GraphPad Software Inc., San Diego, CA, USA)

## 3 Results

### 3.1 Patient and tumour characteristics

Eleven patients with stage IV lung cancer were included in the study. Seven patients with non‐small cell lung cancer (SCLC) and four patients with SCLC were included. Five patients were male, and six patients were females, and the mean age was 66 years old (58–74). All patients completed the study and donated five blood samples each. The patient characteristics are presented in Table 1.

**Table 1:** Patient characteristics. SD, stable disease; PR, partial response.

Patient ID	Histology	Time since biopsy (days)	Time since last treatment (days)	Time since last CT scan (days)	Response
1	Adeno	537	286	27	SD
2	Squamous	656	33	13	SD
3	Small cell	501	32	11	SD
4	Squamous	617	183	7	SD
5	Small cell	298	27	6	PR
6	Small cell	448	53	28	SD
7	Small cell	194	69	48	PR
8	Adeno	191	34	12	PR
9	Adeno	907	32	82	SD
10	Squamous	624	34	20	SD
11	Adeno	133	35	22	PR

Overall, tumour‐specific mutations were identified in the tissue and/or plasma samples from nine out of the eleven patients (82%). The mutation status from the diagnostic biopsy was available in eight of the patients. Plasma DNA was examined using NGS for the remaining three patients. A tumour‐specific mutation was identified in the plasma DNA in one patient with allele frequencies between 67% and 80%. In this specific case, we examined the buffy coat for the identified mutation and no mutation was detected. *TP53* mutations were most frequently found (*n* = 6) followed by *KRAS* mutations (*n* = 3). The mutation characteristics are presented in Table S2.

### 3.2 Intra‐individual variation

Droplet digital PCR was used to evaluate the variation of the mutant allele concentrations. Plasma samples from the nine patients with identified tumour‐specific mutations were analysed using ddPCR. We chose to quantify the tumour‐specific mutation with the highest allele frequency, meaning that only one mutation was quantified for each patient. The identified tumour‐specific mutant alleles were traceable using ddPCR in five patients out of the nine patients with established tumour‐specific mutations. The ddPCR analysis did not retrieve the tumour‐specific mutations in the plasma of the remaining four patients; hence, data on mutation concentration variation were unavailable (Table S3).

**Figure 1:**
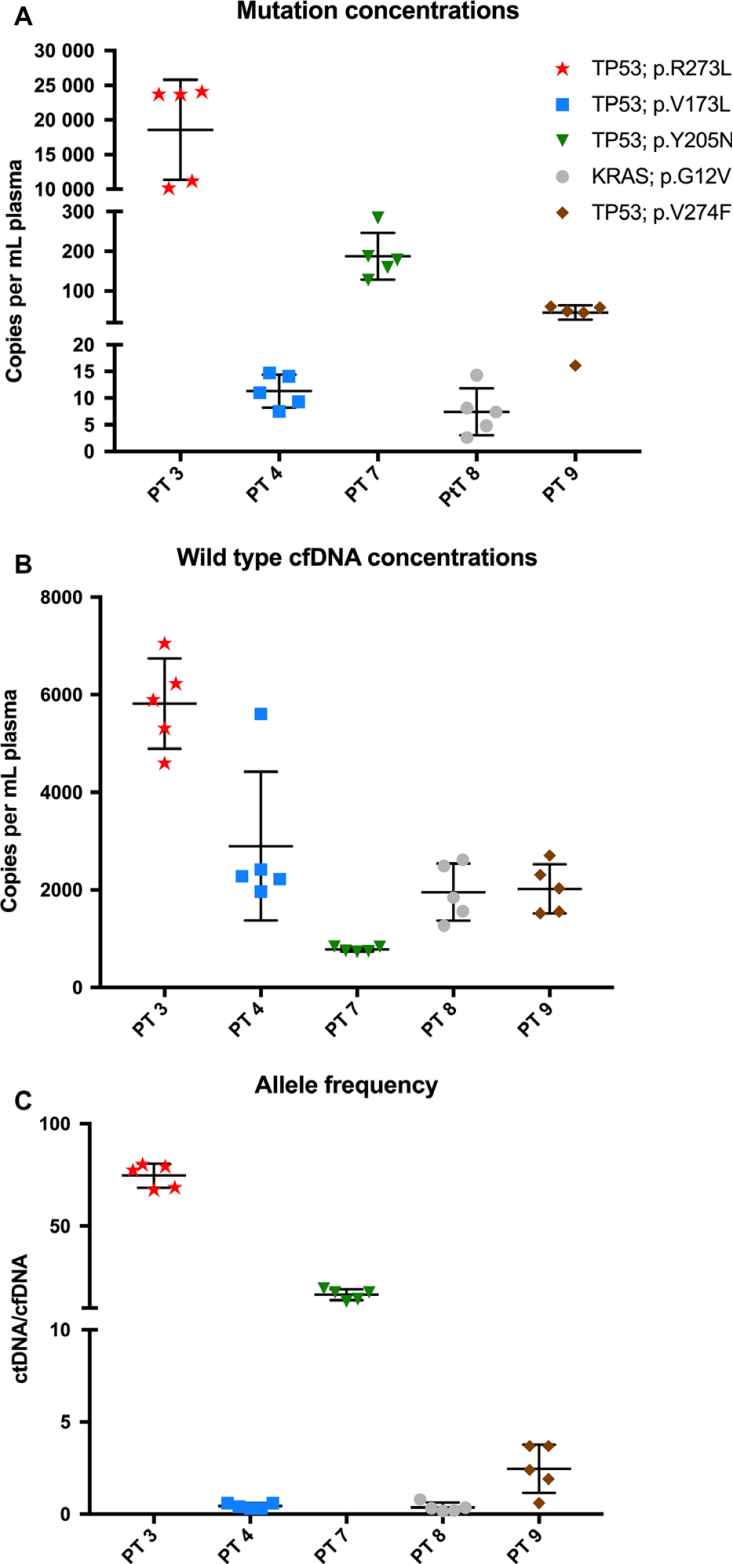
Plasma concentration (copies·mL^−1^) of (A) mutant alleles, (B) wild‐type cfDNA and (C) allele frequencies (ctDNA/cfDNA) in the five patients with detectable mutations by ddPCR. Bars represent mean with standard deviation.

The mean concentration of mutant alleles in the 25 samples from the five patients with traceable mutations was 3769 copies·mL^−1^ and ranged from 4.8 to 24 116 copies·mL^−1^. The mean intra‐individual variation of mutant alleles was 39.1% (range: 27.2–59.3%; Fig. 1A). The wild‐type cfDNA concentration ranged from 731 to 7054 copies·mL^−1^ in the five patients, and the mean intra‐individual variation of the wild‐type cfDNA in all the samples was 26% (range: 7–53%; Fig. 1B). The mean intra‐individual variation of the allele frequency was 36% (range: 8–69%; Fig. 1C).

**Figure 2:**
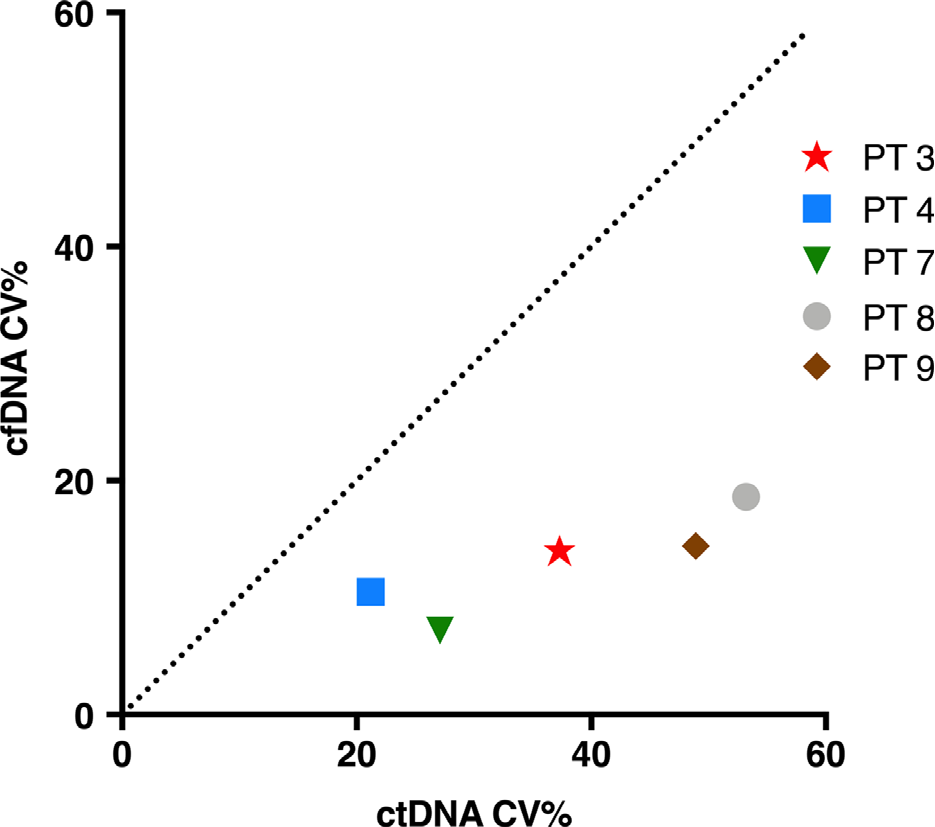
Correlation between CV% of mutant allele concentration and CV% of cfDNA in the five patients with available ddPCR results.

The variation of mutant alleles did not persistently correlate with the cfDNA variations as demonstrated in Fig. 2.

The eluates were tested with a Bioanalyzer to exclude content of high‐molecular‐weight DNA indicating chromosomal contamination from leucocytes. None of the samples contained DNA larger than 160 bp.

### 3.3 Day‐to‐day variation

**Figure 3:**
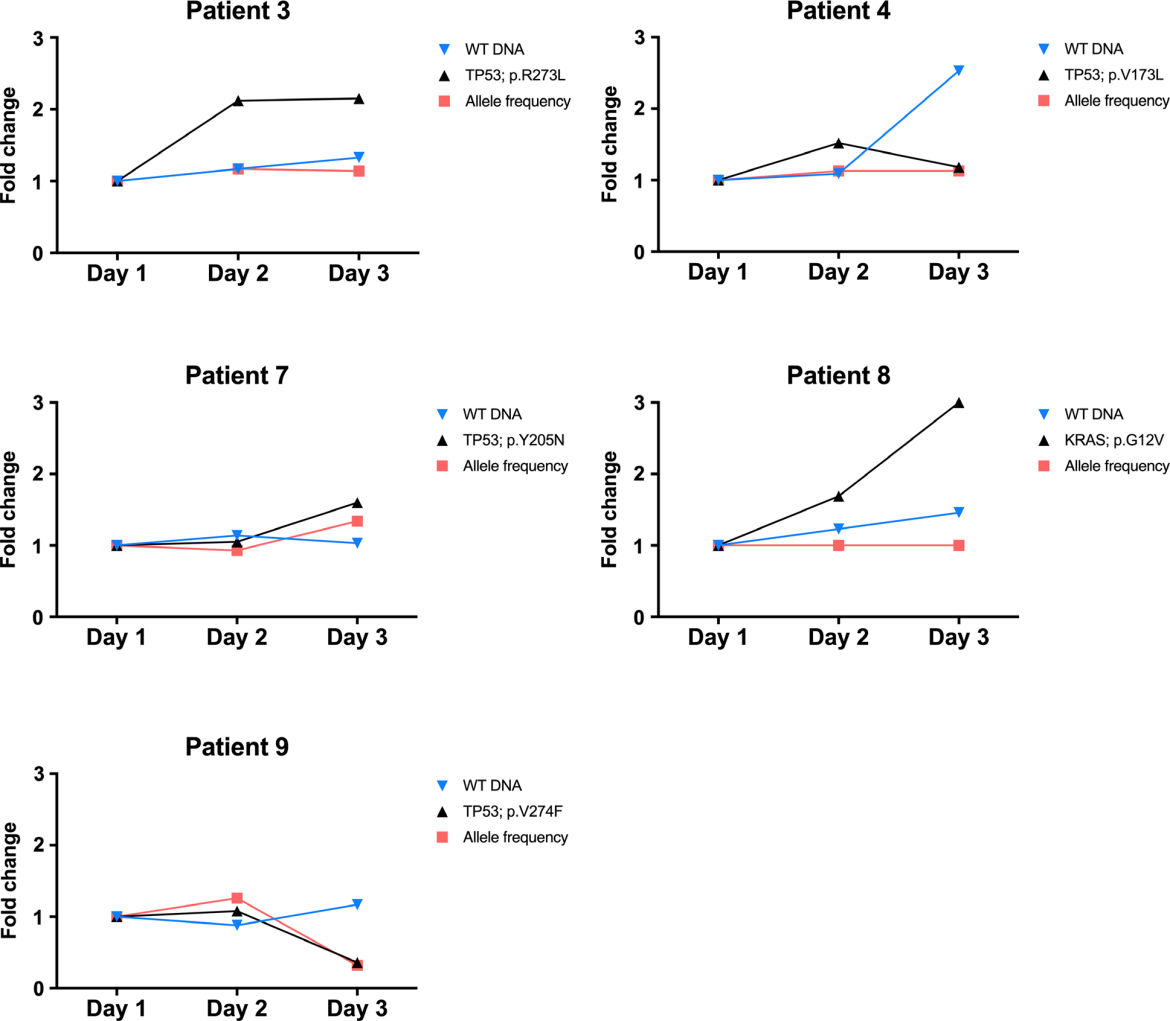
Fold changes of mutant allele concentration, wild‐type DNA and allele frequency from day‐to‐day adjusted to day 1 in the five patients with available ddPCR results.

Since the overall variation within the patient was between 27% and 59%, the day‐to‐day variation in the three samples taken with 24‐h interval was calculated. Intra‐individual day‐to‐day variation of mutant allele concentration was observed in all five patients and ranged between 21% and 53% (Fig. 3; Table 2). Likewise, the day‐to‐day variation of the wild‐type cfDNA was present in all five patients and ranged from 7% to 19% (Table 2) resulting in day‐to‐day variation of the allele frequency between 8% and 74% (Table 2).

**Table 2:** Day‐to‐day variation.

Mutant allele (copies·mL^−1^)					Wild‐type cfDNA (copies·mL^−1^)				Allele frequency			
Pt ID	Day 1	Day 2	Day 3	CV (%)	Day 1	Day 2	Day 3	CV (%)	Day 1	Day 2	Day 3	CV (%)
3	11222.5	23742.9	24116.4	37.3	5318.7	6230.7	7054.2	14	67.8	79.2	77.4	8.2
4	9.3	14.1	11	21.2	2216.9	2420.7	1960.2	10.5	0.4	0.6	0.6	21.7
7	177.9	187.4	284	27.1	731.1	836.4	753.4	7.2	19.6	14.3	17.6	15.6
8	4.8	8.1	14.3	53.2	1265.3	1560.7	1843.3	18.6	0.2	0.3	0.8	73.8
9	45.2	48.9	16.1	48.9	2308.5	2030.7	2701.9	14.4	1.9	2.2	0.6	56.9
Mean CV (%)				37.5	Mean CV (%)			12.9	Mean CV (%)			35.2

The variance of mutated alleles and cfDNA was parallel in the three patients. However, in patient three and patient eight, the allele frequency remained stable despite an increased mutation concentration (Fig. 3).

### 3.4 Hour‐to‐hour variation

Two blood samples (sample 1 and sample 2) were collected with a 1‐h interval on the first and second day of the study. The hour‐to‐hour variation of mutant alleles and the wild‐type cfDNA was calculated using the four blood samples collected on the two successive days. A CV was calculated for each day, and the mean values are listed in Table 3. The mean hour‐to‐hour variation of mutant alleles was 14.1% (range: 0.1–42%), and the mean hour‐to‐hour variation of wild‐type cfDNA was 20.3% (range: 0.1–56%). These variations resulted in hour‐to‐hour variation of the allele frequency between 1% and 47%.

**Table 3:** Hour‐to‐hour variation.

Pt ID	Mutant allele (copies·mL^−1^)				Wild‐type cfDNA (copies·mL^−1^)			Allele Frequency (ctDNA/cfDNA)		
	Day	Sample 1	Sample 2	CV (%)	Sample 1	Sample 2	CV (%)	Sample 1	Sample 2	CV (%)
3	1	11222.0	10177.4	6.9	5318.7	4599.4	10.3	67.8	68.9	1.1
	2	23742.9	23706.1	0.1	6230.7	5897.3	3.9	79.2	80.1	0.8
4	1	9.3	7.5	15.2	2216.9	2279.8	2.0	0.4	0.3	20.2
	2	14.1	14.7	3.0	2420.7	5606.0	56.1	0.6	0.3	47.1
7	1	177.9	159.0	7.9	731.1	745.2	1.4	19.6	17.6	7.6
	2	187.4	127.1	27.1	836.4	834.9	0.1	14.3	13.2	5.7
8	1	4.8	2.6	42.0	1265.3	2487.2	46.1	0.19	0.17	7.9
	2	8.1	7.4	6.4	1560.7	2616.0	35.7	0.31	0.35	8.6
9	1	45.2	60.3	20.2	2308.5	1556.1	27.5	1.9	3.7	45.5
	2	48.9	58.0	12.0	2030.7	1519.8	20.4	2.4	3.7	30.1
		Mean CV(%)		14.1	Mean CV(%)		20.3	Mean CV		17.5

## 4 Discussion

This prospective study investigated the day‐to‐day and hour‐to‐hour variation of ctDNA and wild‐type cfDNA in lung cancer patients with nonprogressive disease.

We demonstrated pronounced intra‐individual day‐to‐day and hour‐to‐hour variation of both ctDNA and wild‐type cfDNA. These variations of ctDNA and wild‐type cfDNA were present at both high and low ctDNA concentrations. Astoundingly, we showed a mutant allele variation of 37% in a patient with a mean mutant allele concentration of 19.694 copies·mL^−1^. Still, as expected, the greatest relative variations of ctDNA were observed in patients with low DNA concentrations.

A great challenge in ctDNA monitoring is defining the accurate number of mutant alleles present in the sample. In previous studies, there has been a great discrepancy in reporting plasma genotyping results. ctDNA results can be presented as either the allele frequency or copies per mL of plasma. We found that variations in mutation concentration did not consistently follow the wild‐type cfDNA variations. Hence, the divergent fluctuation will thereby affect allele frequencies (percentage of ctDNA in proportion to the amount of total DNA in the sample).

These results emphasise that by reporting only allele frequency, results can be misleading. Consequently, using total cfDNA to calculate the allele frequency could add more variation to the result. This underlines the necessity to report both the allele frequencies and total copy numbers per mL when reporting quantitative ctDNA results. Allele frequencies have been used in order to adjust the results to the total amount of DNA in the sample. Looking only at the mutated copies per mL could also be misleading due to the varying DNA isolation efficiency, thereby varying input in the analysis. Hence, one approach could be to report the results as both copies per mL and as allele frequencies when reporting quantitative ctDNA results.

To determine the intra‐individual biological variations in plasma DNA, all external factors with a potential effect on DNA concentration must be eliminated. To ensure that our results would primarily reflect the biological variation of the mutant allele, the potential pre‐analytical bias was kept to a minimum. All the blood samples were processed after exactly 30 min, and the plasma was subsequently frozen at −80 °C. All ddPCR products were conducted in triplicate as a minimum to diminish the variation of the PCR. All five samples from one patient were conducted in the same run, with an intra‐run variability of only 3%. Furthermore, the CV of the extraction was < 10%, which results in an analytical CV well below the DNA variations observed in the patients. Likewise, the fact that the mutated alleles and wild‐type cfDNA varied in different directions in some patients substantiates that the observed variations were biological. If the variation was caused by differences in DNA extraction, the direction of the ctDNA and wild‐type cfDNA variations would be parallel.

To minimise the risk of variation within the patient being influenced by factors other than cancer activity, the patients were instructed to avoid physical activity in connection with blood drawing. Also, patients with infections were excluded. These restrictions were placed because activity and infection are known to affect cfDNA levels (Haller *et al.*, 2017; Moreira *et al.*, 2010). Consequently, every effort has been made so that the demonstrated variations in this study are primarily caused by the metabolism of cancer.

Droplet digital PCR is a highly sensitive DNA quantification method that is optimal for the detection of rare mutation events in an immense background of wild‐type DNA (Hindson *et al.*, 2011). Therefore, ddPCR was the method of choice when analysing the serial samples. In order to use ddPCR to quantify ctDNA, it was essential to identify a tumour‐specific mutation for every patient. In eight of the cases, the mutation status determined from the diagnostic biopsy was available but more than a year had passed since diagnosis for five of these patients. This prolonged time span from the biopsy to inclusion could be the reason for the negative ddPCR results because the patients had received chemotherapy prior to inclusion. This could result in cancer‐specific mutations being below the detection limit at the time of blood sampling. This mutation rate agrees with previous studies, which correspondingly found tumour‐specific mutations in the plasma in ~ 50% of lung cancer patients (Reck *et al.*, 2016; Winther‐Larsen *et al.*, 2017).

In one patient (PT 3), tumour tissue was unavailable. We managed to identify a potential tumour‐specific mutation in plasma by means of NGS. Due to the lack of tissue, we checked the buffy coat for the identified mutation and no mutation was identified. This analysis was conducted to rule out a germline mutation and the risk of clonal haematopoiesis. The negative result verified that the identified mutation was only present in the cancer cells. The remaining four patients with trackable tumour‐specific mutations had tumour tissue available, and we did not perform buffy coat analyses in these four patients. Analyses of the buffy coat in these four patients would have been preferable to rule out clonal haematopoiesis; however, we did not have material available for analysis. Nevertheless, because of the mutation being present in the tumour biopsy, we believe that the mutations being detectable in plasma as a result of clonal hematopoiesis is unlikely.

When the mutation concentration is as low as in patient eight, there is a risk of the variation being caused by subsampling. To minimise the risk of subsampling, we analysed as much material as possible in order to screen as many molecules as possible. Owing to this procedure, we were able to consistently detect tumour‐specific mutations even at very low concentrations.

The EGFR‐mutation status at different sampling points over 1 day has been previously described by Wang *et al. *(2017). They found fluctuating levels but no significant changes in the concentration of EGFR‐mutated ctDNA when they were measured at three different time points. However, in that study the mutated EGFR alleles were sparse, and the results were presented as allele frequencies only. They do not show any information on mutation concentration and wild‐type cfDNA separately, which makes it is difficult to determine whether the registered variation is because of fluctuation of the mutation concentration or caused by wild‐type cfDNA changes.

Our results underline that additional information can be achieved by taking both ctDNA and wild‐type cfDNA variations into consideration.

Due to the limited sample size, we were not able to establish a standard range of intra‐individual ctDNA variation. It would be desirable to determine a precise estimate of the magnitude of ctDNA variations in lung cancer patients with nonprogressive disease, which can only be done in a larger study.

## 5 Conclusion

To the best of our knowledge, this is the first study to address day‐to‐day and hour‐to‐hour variations of ctDNA levels in cancer patients with nonprogressive disease. Our results stress that information on the intra‐individual biological variation of both ctDNA and cfDNA in nonprogressive disease is crucial. Our results on variations of mutant allele concentrations in nonprogressive disease demonstrate that biological variation must be clarified if ctDNA is to be used as a reliable marker of cancer activity in the future.

## Conflict of interest

The authors declare no conflict of interest.

## Author contributions

JAH conceived and designed the study, did data analysis and interpretation of data, laboratory work and wrote the manuscript. ATM designed experiments, did data analysis and interpretation of data, laboratory work and contributed to the composing of the manuscript. HHS designed the clinical study and included patients, and contributed to the composing of the manuscript. SFS conceived and designed the study and contributed to the composing of the manuscript. MS designed experiments, did laboratory work and data analysis, and contributed to the composing of the manuscript. PM designed the clinical study and contributed to the composing of the manuscript. BS designed experiments, interpreted data and contributed to the composing of the manuscript.

## Supporting information


**Table S1** **.** ddPCR assay information.

**Table S2** **.** Mutation characteristics.

**Table S3** **.** ddPCR results.
