# Supplementary material for: Intra‐individual variation of circulating tumour DNA in lung cancer patients
Source: Mol Oncol. 2019 Aug 16;13(10):2098–106. doi: 10.1002/1878-0261.12546 (PMC6763781; doi:10.1002/1878-0261.12546)
Supplement: Supplementary file 1 — Table S1 . ddPCR assay information. [file MOL2-13-2098-s001.docx]

| **Supplemental Table 1: ddPCR assay information** | | | | |  |  |  |  |  |
| --- | --- | --- | --- | --- | --- | --- | --- | --- | --- |
| Locus | Gene | Mutation | | COSMIC ID | Bio-Rad assay ID | Amplicon size (bp) | Annealing temperature (°C) | Validated^1^ | LoD (%) |
|  |  | CDS | Amino acid |  |  |  |  |  |  |
| Chr12:25398284 | KRAS | c.35G>T | p.G12V | COSM520 | dHsaCP2500592, dHsaCP2500593 | 57 | 55 | Yes | 0,1 |
| Chr12:25398285 | KRAS | c.34G>T | p.G12C | COSM516 | dHsaCP2500584, dHsaCP2500585 | 57 | 55 | Yes | 0,3 |
| Chr17:7577058 | TP53 | c.880G>T | p.E294* | COSM10856 | dHsaMDV2516862 | 80 | 55 | Yes | 0,2 |
| Chr17:7577118 | TP53 | c.820G>T | p.V274F | COSM10769 | dHsaMDS2516048 | 65 | 55 | No | 0,1 |
| Chr17:7577120 | TP53 | c.818G>T | p.R273L | COSM10779 | dHsaMDV2510504 | 65 | 55 | Yes | 0,2 |
| Chr17:7578236 | TP53 | c.613T>A | p.Y205N | COSM45685 | dHsaMDS2514224 | 76 | 55 | No | 0,4 |
| Chr17:7578413 | TP53 | c.517G>T | p.V173L | COSM43559 | dHsaMDS2511406 | 64 | 55 | No | 0,2 |
| Chr17:7578454 | TP53 | c.475_476delGC | p.A159fs | COSM87652 | dHsaMDS385448559 | 80 | 55 | No | 0,1 |
| Abbreviations: CDS, coding DNA sequence; del, deletion; *, translation termination; fs, frameshift; bp, base pairs; LoD, limit of detection | | | | | | | | | |
| 1: Validated indicates if the assay has been wet-lab validated by Bio-Rad | | | | | | | | | |
